# Supplementary material for: Towards the Sustainable Exploitation of Salt-Tolerant Plants: Nutritional Characterisation, Phenolics Composition, and Potential Contaminants Analysis of Salicornia ramosissima and Sarcocornia perennis alpini
Source: Molecules. 2023 Mar 17;28(6):2726. doi: 10.3390/molecules28062726 (PMC10059647; doi:10.3390/molecules28062726)
Supplement: Supplementary file 1 [file molecules-28-02726-s001.zip › molecules-2257420-supplementary.pdf]

molecules

MDPI

Supplementary Material

Table S1. Moisture (% fresh weight (fw)), nutrient composition (% dry weight (dw)), minerals (mg/g dw), and total phenolics content (TPC) (mg of gallic acid equivalents (GAE)/g extract dw) of different *Salicornia* species (data compiled from the literature).

**Parameter**	***S. ramosissima***				***S. europaea***	***S. herbacea***			***S. bigelovii***
	Wild; central Portugal [8]	Wild; southern Portugal [12]	Cultivated; southern Portugal [95]	Wild; central Portugal [40]	Wild; northern Serbia [96]	Wild; southwestern Tunisia [14]	Wild; southeastern Tunisia [97]		Cultivated; western United States of America [98]
							Region A	Region B	
**Nutrient composition**									
Moisture	85.62 ± 0.28	84.5 ± 0.2	NA			NA	NA	NA	NA
Crude protein	4.16 ± 0.71	5.20 ± 0.29	NA			22.10 ± 1.28	NA	NA	10.2 ± 1.0
Lipids	0.45 ± 0.10	1.87 ± 0.18	NA			1.70 ± 0.15	2.20 ± 0.18	4.50 ± 0.23	NA
Total carbohydrates	48.0	63.73 [b]	NA			36.17 ± 0.84	NA	NA	NA
Fibre	10.36 ± 0.74 [a]	22.5 [c]	NA			NA	NA	NA	19.0 ± 0.5 [c]
Ash	47.38 ± 0.27	29.2 ± 0.6	NA			8.10 ± 0.36	23.50 ± 0.41	40.93 ± 0.63	52.70 ± 1.41
**Minerals**									
Sodium	159.27 ± 0.76	89.9 ± 0.5	NA			3.33	9.00 ± 2.27	14.2 ± 6.23	169.2 ± 4.4
Potassium	7.22 ± 0.13	8.92 ± 0.23	NA			0.02	1.58 ± 1.56	1.73 ± 1.54	NA
Chloride	237.02 ± 1.35	NA	NA			NA	NA	NA	119.9 ± 20.4
Calcium	2.99 ± 0.00	4.86 ± 0.05	NA			NA	1.52 ± 1.43	1.51 ± 1.26	7.6 ± 1.4
Phosphorous	0.36 ± 0.00	NA	NA			0.31	NA	NA	NA
Magnesium	9.01 ± 0.20	9.43 ± 0.08	NA			TR	0.45 ± 0.34	0.38 ± 1.67	5.8 ± 0.2
**Total phenolics content**	NA	33.0 ± 0.7	25.66 ± 2.22	15.02 ± 2.01	58.20 ± 0.44	53.8 ± 2.6	32.1 ± 0.3	43.1 ± 0.2	NA

[a] Crude fibre determined by Weende method.

[b] Not reported, estimated by difference.

[C] Neutral detergent fibre.

NA – Not analysed; TR – Traces.

Table S2. Moisture (% fresh weight (fw)), nutrient composition (% dry weight (dw)), minerals (mg/g dw), and total phenolics content (TPC) (mg of gallic acid equivalents (GAE)/g extract dw) of different *Sarocornia* species (data compiled from the literature).

Parameter	*S. perennis alpini*	*S. perennis perennis*	*S. fruticosa*			*S. ambigua*		*S. neei*	
	Wild; southern Portugal [12]	Wild; southern Portugal [12]	Wild; southern Spain [11]	Wild; Southern Portugal [11]	Cultivated [11]	Southern Brazil [13]		Central Chile [24]	
						Wild	Cultivated	Wild	Cultivated
**Nutrient composition**									
Moisture	84.0 ± 1.4	85.8 ± 0.5	84.1 ± 0.4	87.0 ± 0.3	92.0 ± 0.1	88.15 ± 0.54	88.57 ± 0.60	89.78 ± 1.30	86.96 ± 1.05
Crude protein	8.10 ± 0.06	6.90 ± 0.17	9.25 ± 0.03	9.55 ± 0.04	12.6 ± 0.62	16.29 ± 0.17	18.02 ± 0.09	13.50 ± 1.76	6.98 ± 0.31
Lipids	1.20 ± 0.09	2.25 ± 0.05	4.45 ± 0.06	4.41 ± 0.05	5.60 ± 0.15	1.35 ± 0.08	1.05 ± 0.79	1.08 ± 0.59	1.07 ± 0.31
Total carbohydrates	60 [a]	67.6 [a]	57.4 [a]	48.7 [a]	38.5 [a]	29.96	27.47	49.61 [a]	56.21 [a]
Fibre	20.8 [b]	34.1 [b]	22.0 ± 0.00 [c]	16.6 ± 0.01 [c]	9.26 ± 0.01 [c]	~23.2 [c]	~17.2 [c]	9.98 ± 3.52 [d]	16.95 ± 1.76 [d]
Ash	30.7 ± 1.1	23.3 ± 0.3	28.9 ± 0.1	37.3 ± 0.0	43.3 ± 0.1	24.98 ± 11.05	31.85 ± 10.59	35.81 ± 1.96	35.74 ± 0.84
**Minerals**									
Sodium	64.3 ± 0.9	64.1 ± 0.9	243 ± 6	253 ± 14	297 ± 4	85.99 ± 44.73	144.97 ± 15.75	80.7 ± 4.7	100.1 ± 6.9
Potassium	10.3 ± 0.1	13.9 ± 0.1	62.4 ± 1.1	49.6 ± 0.5	87.5 ± 1.0	24.47 ± 9.28	15.84 ± 0.87	20.3 ± 0.9	15.5 ± 0.9
Chloride	NA	NA	NA	NA	NA	NA	NA	155.8 ± 7.3	150 ± 19.5
Calcium	2.63 ± 0.01	2.34 ± 0.01	24.7 ± 0.6	21.3 ± 1.1	17.8 ± 0.1	4.56 ± 34.60	4.64 ± 1.75	6.3 ± 0.7	5.3 ± 0.5
Phosphorous	NA	NA	NA	NA	NA	NA	NA	1.8 ± 0.2	1.6 ± 0.2
Magnesium	7.03 ± 0.04	6.73 ± 0.08	59.2 ± 0.7	28.7 ± 0.9	17.4 ± 0.3	7.76 ± 4.22	11.37 ± 0.87	11.3 ± 1.6	8.2 ± 0.8
**Total phenolics content**	20.7 ± 0.5	20.5 ± 0.5	5.69 ± 0.06	5.12 ± 0.07	4.10 ± 0.0	NA	NA	6.13 ± 0.24	2.85 ± 0.24

[a] Not reported, estimated by difference.

[b] Neutral detergent fibre.

[c] Total dietary fibre.

[d] Crude fibre.

NA – Not analysed.
